# Analysis of Fluoride-Free Content on Twitter: Topic Modeling Study

**DOI:** 10.2196/44586

**Published:** 2023-06-20

**Authors:** Matheus Lotto, Irfhana Zakir Hussain, Jasleen Kaur, Zahid Ahmad Butt, Thiago Cruvinel, Plinio P Morita

**Affiliations:** 1 Department of Pediatric Dentistry, Orthodontics and Public Health Bauru School of Dentistry University of São Paulo Bauru Brazil; 2 School of Public Health Sciences University of Waterloo Waterloo, ON Canada; 3 Department of Data Science and Business Systems, School of Computing College of Engineering and Technology SRM Institute of Science and Technology Kattankulathur India; 4 Research Institute for Aging University of Waterloo Waterloo, ON Canada; 5 Department of Systems Design Engineering University of Waterloo Waterloo, ON Canada; 6 eHealth Innovation Techna Institute University Health Network Toronto, ON Canada; 7 Institute of Health Policy, Management, and Evaluation Dalla Lana School of Public Health University of Toronto Toronto, ON Canada

**Keywords:** fluoride, health information, infodemiology, infoveillance, misinformation, social media, Twitter, oral care, healthy lifestyle, hygiene

## Abstract

**Background:**

Although social media has the potential to spread misinformation, it can also be a valuable tool for elucidating the social factors that contribute to the onset of negative beliefs. As a result, data mining has become a widely used technique in infodemiology and infoveillance research to combat misinformation effects. On the other hand, there is a lack of studies that specifically aim to investigate misinformation about fluoride on Twitter. Web-based individual concerns on the side effects of fluoridated oral care products and tap water stimulate the emergence and propagation of convictions that boost antifluoridation activism. In this sense, a previous content analysis–driven study demonstrated that the term *fluoride-free* was frequently associated with antifluoridation interests.

**Objective:**

This study aimed to analyze “fluoride-free” tweets regarding their topics and frequency of publication over time.

**Methods:**

A total of 21,169 tweets published in English between May 2016 and May 2022 that included the keyword “fluoride-free” were retrieved by the Twitter application programming interface. Latent Dirichlet allocation (LDA) topic modeling was applied to identify the salient terms and topics. The similarity between topics was calculated through an intertopic distance map. Moreover, an investigator manually assessed a sample of tweets depicting each of the most representative word groups that determined specific issues. Lastly, additional data visualization was performed regarding the total count of each topic of fluoride-free record and its relevance over time, using Elastic Stack software.

**Results:**

We identified 3 issues by applying the LDA topic modeling: “healthy lifestyle” (topic 1), “consumption of natural/organic oral care products” (topic 2), and “recommendations for using fluoride-free products/measures” (topic 3). Topic 1 was related to users’ concerns about leading a healthier lifestyle and the potential impacts of fluoride consumption, including its hypothetical toxicity. Complementarily, topic 2 was associated with users’ personal interests and perceptions of consuming natural and organic fluoride-free oral care products, whereas topic 3 was linked to users’ recommendations for using fluoride-free products (eg, switching from fluoridated toothpaste to fluoride-free alternatives) and measures (eg, consuming unfluoridated bottled water instead of fluoridated tap water), comprising the propaganda of dental products. Additionally, the count of tweets on fluoride-free content decreased between 2016 and 2019 but increased again from 2020 onward.

**Conclusions:**

Public concerns toward a healthy lifestyle, including the adoption of natural and organic cosmetics, seem to be the main motivation of the recent increase of “fluoride-free” tweets, which can be boosted by the propagation of fluoride falsehoods on the web. Therefore, public health authorities, health professionals, and legislators should be aware of the spread of fluoride-free content on social media to create and implement strategies against their potential health damage for the population.

## Introduction

Social media have increasingly gained popularity as platforms for individuals and communities to seek and share health information. In that regard, Twitter boasts an impressive user base of approximately 368 million monthly active individuals who share a common interest in discussing common health topics, such as COVID-19, cancer, influenza, mental health, vaccination, and smoking [1-7]. Although Twitter can be incredibly informative and engaging for health seekers, it can also be a breeding ground for misinformation [5]. In this context, misinformation indicates an umbrella term covering a range of false or misleading content, such as misinformation, mal-information, disinformation, fake news, and conspiracy theories [8-12].

Although Twitter can contribute to spreading health falsehoods, it can also serve as a valuable tool for elucidating the social factors that contribute to the onset of negative beliefs. As a result, data mining has become a widely used technique in infodemiology and infoveillance research to combat misinformation effects [1,13,14]. For example, screening COVID-19 vaccine misinformation on Twitter helped to identify the health beliefs associated with the antivaccination movement, which contributed to a better understanding of the factors relating to the prevalence and severity of cases in different countries [15]. Moreover, the analysis of social media content can contribute to the development of artificial intelligence–based ecosystems to control the misinformation phenomenon [16,17]. Previously, content analysis on social media required manual characterization, but now, machine learning models can also be used to analyze large data sets.

Since its introduction in 1942, multiple studies have consistently demonstrated the role of fluoride in reducing the prevalence and incidence of caries lesions [18]. There is robust evidence that fluoride interventions, including the fluoridation of tap water, the use of fluoride-containing oral care products, and professional fluoride application, promote favorable health outcomes [19-22]. The prevalence of dental caries has decreased drastically among people from communities exposed to these preventive, cost-effective measures [20,23]. One concern surrounding fluoride use is that overexposure, specifically during tooth development stages, increases the chance of dental fluorosis in children [24]. Thus, dentists recommend the adoption of safeguard measures for the storage and use of fluoride-containing products, such as keeping them in a secure place out of children’s reach, reducing the amount of toothpaste during tooth brushing, and avoiding fluoridated solutions with pleasant flavors [19,25]. This safety consideration, in conjunction with misinformation that fluoride causes neurological disorders, has flooded the internet with erroneous beliefs on the poisoning capacity of fluoride [26,27]. This spread of misinformation has contributed to a public refusal to use fluoride to control dental caries [16,28]. Specifically, a previous content analysis–driven study demonstrated that the term *fluoride-free* was frequently associated with antifluoridation interests on social media [16].

In view of possible negative implications of web-based fluoride content, this study aimed to analyze “fluoride-free” tweets regarding their topics and frequency of publication over time.

## Methods

### Ethical Considerations

This study did not require institutional review board approval from the Council of Ethics in Human Research of the University of Waterloo because federal regulations do not apply to research using publicly available data that do not involve human subjects.

### Data Collection

On June 5, 2022, the Twitter application programming interface [29] was used to extract 23,436 tweets published in English between May 2016 and May 2022 with the keyword “fluoride-free.” It followed the parameters of a prior study about fluoride-related misinformation on Instagram [16].

### Preprocessing of the Data Set

The CSV file data set was uploaded and processed to enhance the quality of the tweets for subsequent analysis [30]. Initially, duplicate tweets were identified and eliminated, resulting in a data set containing 21,169 unique tweets. Next, tokenization was performed to break down the text into smaller units, such as individual words. Then, all tokens were converted to lowercase to ensure uniform treatment, irrespective of capitalization, and to prevent potential discrepancies in the analysis. Stop words were removed to eliminate common words that contribute little meaning to the analysis. This included articles (eg, “a,” “an,” and “the”), prepositions (eg, “on,” “in,” and “at”), and conjunctions (eg, “and,” “but,” and “or”). Additionally, unspecific or irrelevant words were removed, such as non-English characters, keywords, verbs, and digits. Lastly, punctuation marks, symbols, and special characters were eliminated to refine the data set further and ensure consistency in subsequent analysis.

Furthermore, exploratory data preprocessing was performed to assess the impact of both the most frequent and rare words in the data set. Notably, term frequency filtering did not exhibit substantial improvements on latent Dirichlet allocation (LDA) topic modeling. Therefore, the decision was made to retain the most frequent and rare words to enhance the contextual relevance of the resulting topics (Multimedia Appendix 1).

### Data Analyses

LDA topic modeling is an unsupervised machine learning probabilistic and word count–based algorithm used to effectively determine salient terms and extract emerging groups (topics) of a particular corpus (data set), including social media posts concerning health issues [31,32]. Even though this analysis does not provide a complete meaning of social media posts, it contributes to a good overview of their main issues [33]. Notably, a detailed description of the LDA model is provided elsewhere [34].

The preprocessed 21,169 tweets were subjected to LDA topic modeling techniques using the Python-based *Gensim* and *spaCy* libraries in a Google Colaboratory interface to identify the salient terms and topics [31,32]. The number of ideal topics was defined according to the coherence score proposed by Nikolenko and colleagues [35].

In this context, the coherence values were computed for *K* topics, where *K* ranged from 2 to 50, before eventually narrowing the consideration range down to 3 to 15 cases. Remarkably, higher coherence scores probabilistically represent better-quality topic modeling, facilitating the interpretation of outputs. An investigator (ML) validated the topic model with the highest coherence score by rating the model on the meaningfulness of its topics, how well publications within a single topic are related, and how publications of different topics are distinct.

Facing the definition of the number of topics, the similarity between them was calculated through an intertopic distance map. It is a visualization of the issues in a bidimensional space, where the bubbles represent topics that emerged from the topic modeling analysis and are dimensionally proportional to the number of contained words. In this graph representation, the axes are based on 2 principal components to facilitate the interpretation of the distance between topics. Synthetically, the LDA algorithm uses a principal component analysis to reduce N-dimensional vectors to bidimensional vectors and achieve (*x,y*) coordinates.

Moreover, the same investigator (ML) manually assessed a sample of tweets depicting each of the most representative word groups that determined specific issues. Lastly, additional data visualization was performed regarding the total count of each topic of fluoride-free record and its relevance over time, using Elastic Stack software through the Elasticsearch and Kibana features [36-38].

## Results

**Figure 1:**
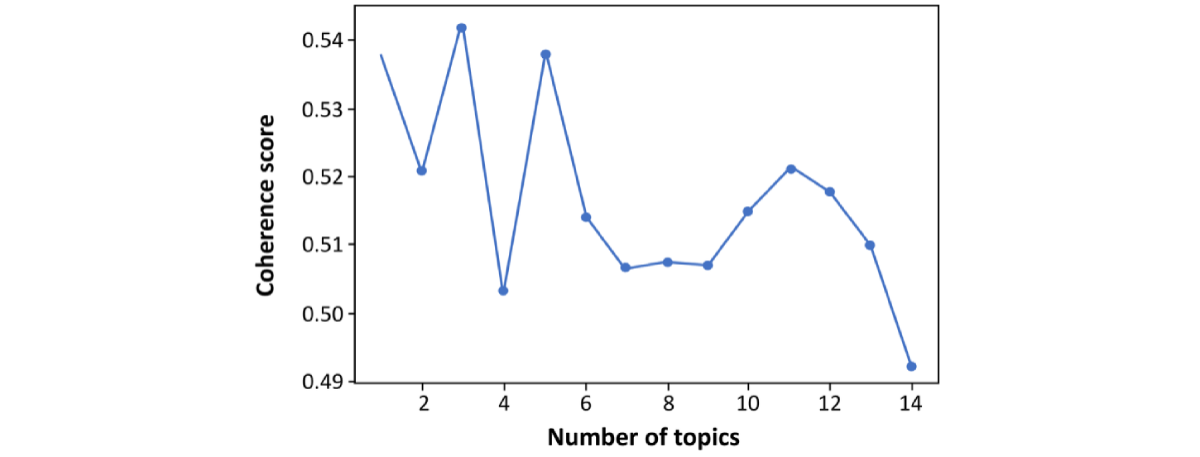
Coherence scores for distinct number of topics.

To determine the underlying social issues surrounding the spread of fluoride-free content on Twitter, we applied topic modeling to identify patterns based on the frequency of keywords [29,30]. A higher coherence score is associated with better data quality and simplified output interpretation. The highest coherence value (0.543) occurred for 3 topics (Figure 1). This indicated that the Twitter posts contained 3 different fluoride-free–related topics.

**Figure 2:**
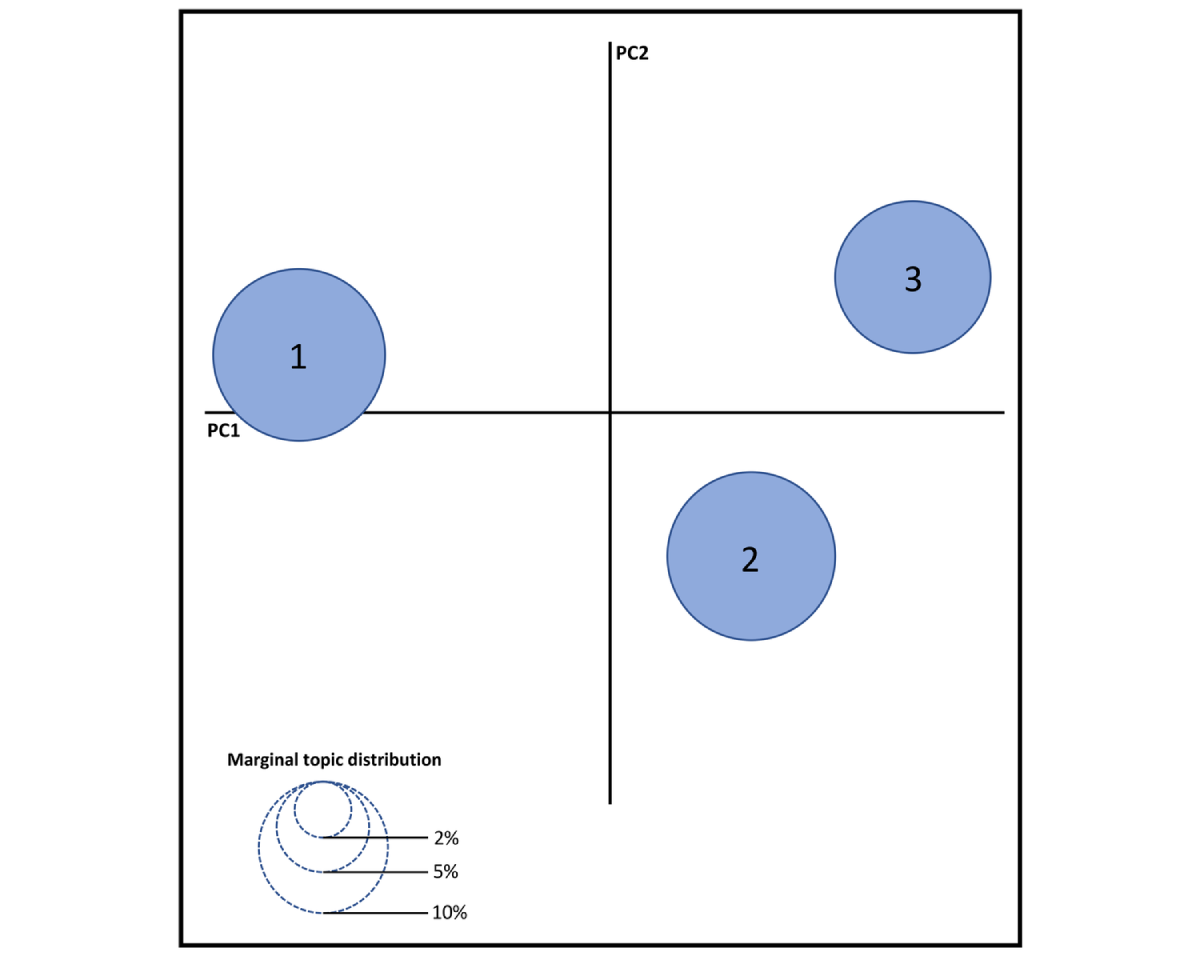
Intertopic distance map. The bubbles are denominated according to the number of the specific topic. PC: principal component.

Using an intertopic distance map (Figure 2), we visualized the arrangement of topics based on the words they comprise. The analysis revealed a distance between the topics, confirming the identification of 3 distinct issues: “healthy lifestyle” (topic 1), “consumption of natural/organic oral care products” (topic 2), and “recommendations for using fluoride-free products/measures” (topic 3). In this sense, topic 1 was related to users’ concerns about leading a healthier lifestyle and the potential impacts of fluoride consumption, including its hypothetical toxicity. Complementarily, topic 2 was associated with users’ personal interests and perceptions of consuming natural and organic fluoride-free oral care products, whereas topic 3 was linked to users’ recommendations for using fluoride-free products (eg, switching from fluoridated toothpaste to fluoride-free alternatives) and measures (eg, consuming unfluoridated bottled water instead of fluoridated tap water), comprising the propaganda of dental products.

Table 1 summarizes the fluoride-free salient topics stratified according to the most frequent words, issues, and examples. It is important to note that some words appear in 2 topics, for example, “tooth” in topics 2 and 3. The homogeneity of the data set can explain this pattern since tweets were collected from a keyword that restricts the results to a specific theme. Moreover, some words per se do not directly represent the issue of the topic, such as “long” or “black” in topic 1 (healthy lifestyle). Thus, the context of tweets is essential to define the issue of emerging topics in both cases. As a result, the analysis of a sample of tweets from each topic was necessary to ensure the distinction between issues presented within the topics.

**Figure 3:**
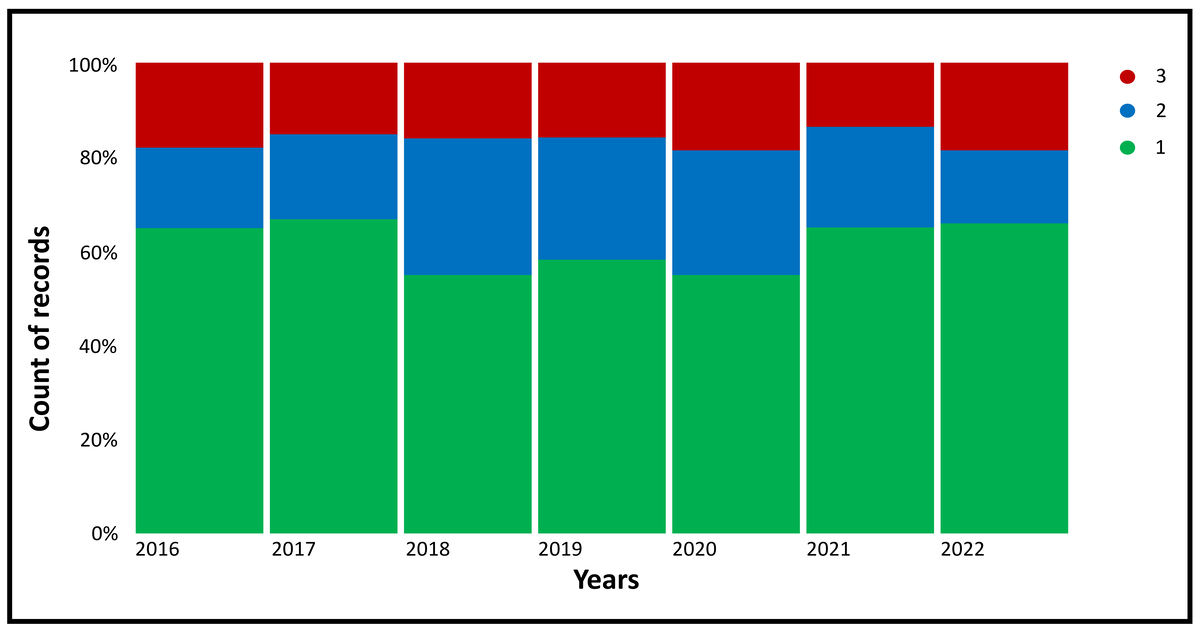
Percentage of tweets related to each topic from latent Dirichlet allocation analysis over time. Green bars represent topic 1 (healthy lifestyle), blue bars represent topic 2 (consumption of natural/organic oral care products), and red bars represent topic 3 (recommendations for using fluoride-free products/measures).

**Figure 4:**
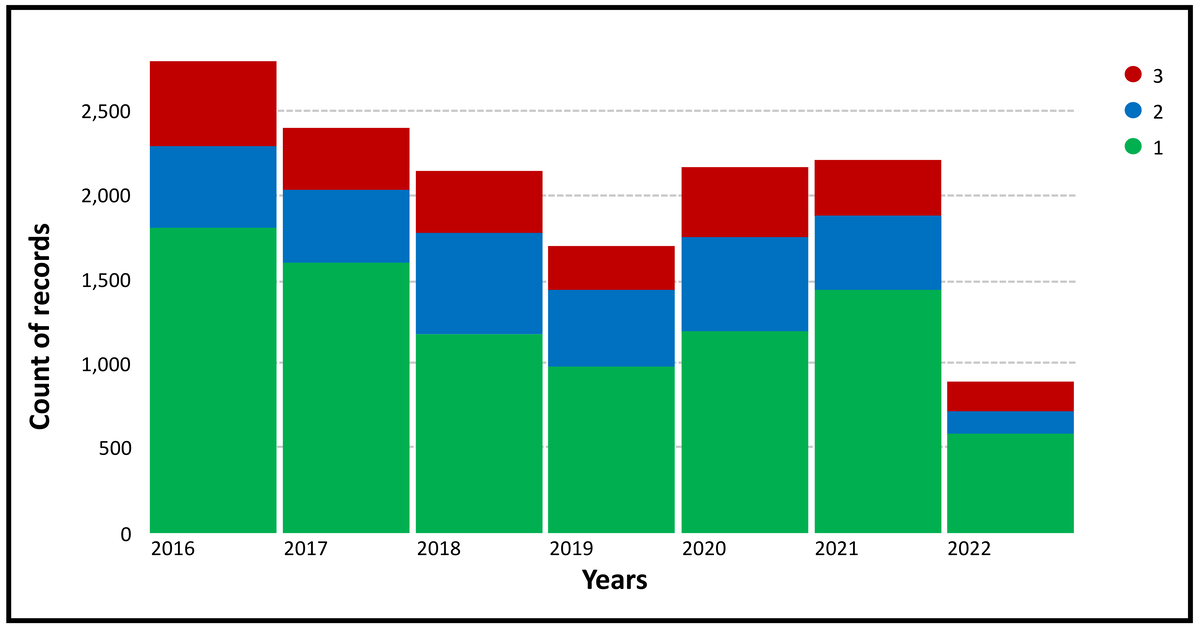
Number of tweets related to each topic from latent Dirichlet allocation analysis over time. Green bars represent topic 1 (healthy lifestyle), blue bars represent topic 2 (consumption of natural/organic oral care products), and red bars represent topic 3 (recommendations for using fluoride-free products/measures). Note that the lower count observed in 2022 is relative to the partial data collection until May (less than half year).

To determine the frequency that each “fluoride-free” topic appears on Twitter, we calculated the percentage (Figure 3) and absolute count (Figure 4) of each topic over time. Topic 1 (healthy lifestyle) represents the largest share of tweets, whereas topics 2 (consumption of natural/organic oral care products) and 3 (recommendations for using fluoride-free products/measures) are similar in terms of their representation (Figure 3). Between 2016 and 2019, a gradual decline in “fluoride-free” tweets was observed, reaching <2000 tweets per year (Figure 4). Nonetheless, the number of tweets increased again from 2020 onward.

**Figure 5:**
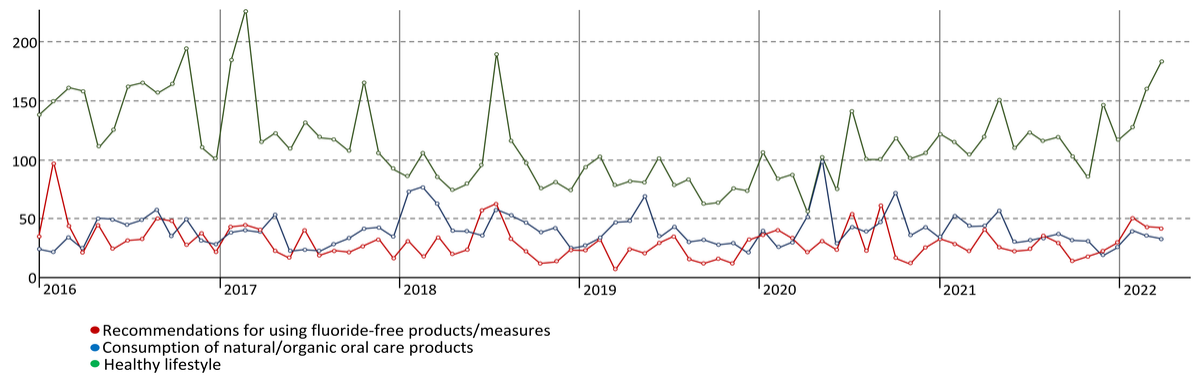
The relevance of topics over time (2016-2022). Note that each point corresponds to a 4-week interval.

The relevance of each topic was determined by the number of tweets over time. The points of each curve are separated by a 4-week interval (Figure 5). The number of tweets from topic 1 (healthy lifestyle) was higher than other topics, particularly in 2016, 2017, 2021, and 2022. The relevance of topics 2 (consumption of natural/organic oral care products) and 3 (recommendations for using fluoride-free products/measures) was similar over time, with a slight advantage for topic 2 in 2018.

**Table 1:** Fluoride-free–related salient topics stratified according to the most frequent words, issues, and examples.

Topic	Most frequent words	Issues	Examples
1	Natural, oil, product, vegan, organic, charcoal, people, black, care, kid, family, long, food, deodorant, good, and dentist	Healthy lifestyle	“Chlorine free. Fluoride-free. We believe that a better you start with a better water.” “Fluoride-free health products to support a healthy lifestyle”
2	Tooth, good, dental, low, health, paste, organic, people, Tom, well, aluminum, child, deodorant, cavity, year, and way	Consumption of natural/organic oral care products	“Sprouts has very good vegan lip balm, lotion, deodorant, natural oils, fluoride-free and all natural tooth paste etc.” “Anybody know of a site that does that organic fluoride free toothpaste, aluminum free”
3	Tooth, Tom, decay, oil, state, week, health, Maine, dentist, new, deodorant, oral, low, whitening, cavity, safe, and day	Recommendations for using fluoride-free products/measures	“Fluoride does a body bad. Don‘t drink fluoride. I like to use Tom‘s fluoride-free toothpaste.” “Tom’s of Maine natural fluoride-free baking soda toothpaste with propolis and myrrh”

## Discussion

### Principal Findings

The purpose of this study was to analyze the fluoride-free content on Twitter. Although studies have investigated fluoride-related content on Facebook, Instagram, and Twitter [16,28,39-42], to the best of our knowledge, this is the first study that focused on fluoride-free content on Twitter using topic modeling methods. The findings demonstrated that fluoride-free content found on Twitter mainly concerned a perceived healthy lifestyle, followed by the consumption of natural and organic oral care products and recommendations of fluoride-free products and measures. The outcomes are in accordance with previous studies [16,28,39-42]. We found that the interest in falsehoods gradually decreased between 2016 and 2019 and subsequently rose again from 2020 onward. The use of topic modeling analysis enabled the examination of a large Twitter data set, which would be difficult to achieve with traditional human-based analysis. In this sense, developing artificial intelligence–grounded systems capable of detecting and analyzing a massive volume of fluoride-related misinformation is essential.

Despite a decline in “fluoride-free” tweets between 2016 and 2019, the antifluoridation movement has had a negative impact on dental public policies in English-speaking countries. This has led to some regions discontinuing community water fluoridation to meet specific demands of users, despite community-based intervention being a key factor in promoting oral health equity [43]. For example, in Canada, only approximately 39% of the population has access to fluoridated tap water, with uneven distribution across the country (71% living in Ontario vs 1.7% living in British Columbia) [44]. Research has shown that dental caries prevalence is significantly higher in areas where fluoridation has been discontinued. An observational study conducted on schoolchildren in Alberta found that dental caries prevalence in primary dentition (ie, deciduous teeth and baby teeth) was significantly higher in Calgary (64.8%; fluoridation cessation in 2011) compared to Edmonton (55.1%; still fluoridated) [45]. In contrast, oral care companies have developed fluoride-free products to meet consumers’ preferences [16,46]. Interestingly, the increase in “fluoride-free” tweets in 2020 coincided with the onset of the COVID-19 pandemic. The infodemic scenario during the pandemic has led to increased health information-seeking behavior among users, including dental-related issues [47,48].

False health beliefs usually originate from the same mechanisms of accurate health beliefs, influenced by diverse cognitive, social, and affective drivers such as intuitive thinking (the absence of analytical thinking or deliberation), cognitive failures (neglecting or forgetting sources and evidence), illusory truth (familiarity, fluency, and cohesion), reliance on source cues (elites, in-group, and attractive), emotion (emotive content and emotional state), and worldview (personal views and partisanship) [49,50]. Notably, users tend to uncritically interact with posts associated with their health beliefs, reinforcing the genesis of echo chambers on social media [51,52]. This scenario propitiates the emergence of denialist groups, characterized by their high level of engagement with health misinformation publications. In this context, anti–community water fluoridation groups’ digital activity exceeds those of their counterparts by 60 times [40]. In addition, developing health beliefs have a significant association with lifestyle and impacts on its changes [53], which could explain the high demand for this topic in our study.

### Practical Implications

The findings may contribute to elaborating educational public health actions regarding eHealth and mobile health solutions, aiding communities in avoiding antifluoridation content on social media. Likewise, these results can orient fact-checking agencies to promptly clarify deceitful web-based publications about fluoride to their audience, addressing the concerns of users to support fluoride-containing measures. For example, issues concerning fluoride overexposure must be clarified, reinforcing safeguard actions for storing and using fluoridated products in early childhood [19,25]. Certainly, the adoption of reliable web-based health information by users can enhance individual and shared decision-making, thus contributing to better health care outcomes [54,55]. In this context, government agencies and organizations should be aware of the importance of investments in measures designed to improve the acquisition of information by the population [56,57]. Moreover, artificial intelligence–based systems may support public health actions to mitigate the propagation of false fluoride content on the web. Specifically, these solutions can automatically label publications and down-promote them until an in-depth investigation by social media companies. With insight into the underlying social media issues surrounding antifluoridation content, health professionals can ensure adequate communication with their patients based on person-centered care principles [58]. Equally important, legislators must discuss resolutions to control the spread of misinformation, specifically the role and duties of users and social media managers. Since the antifluoridation movement is substantially present in contemporary society, fluoride refusal will frequently be reported in dental offices [28].

### Limitations

This study has some limitations. First, we used only data from Twitter for the proposed analyses. As a result, the findings are influenced by platform characteristics (eg, limitation in the word count of posts), differentiating them from other social media. Despite that limitation, previous studies also focused on Twitter data to investigate health topics on social media because of the advantages relating to data collection and the number of active users [13,59]. Second, we collected the Twitter data set from a specific keyword, which may have limited the extrapolation of outcomes to all antifluoridation content. Nevertheless, we defined the search strategy and collected the data based on a previous fluoride misinformation study [16]. Third, although the results indicate the need to control information disorder on Twitter more effectively, it is improbable that the company will modify its policies in this area due to recent changes in ownership. Fourth, trend analysis did not consider the influence of possible platform-related confounders, such as the increase or decrease in the overall number of tweets on Twitter or changes in the content moderation policy over time. However, trend patterns are likely more related to users’ interest in fluoride-free content than Twitter’s characteristics. Lastly, these findings were based only on English-language tweets, influencing the screening of fluoride-free content by cultural and regional aspects. In contrast, falsehoods tend to be found in English since it is the most spoken language in the world.

### Conclusions

The public concerns toward a healthy lifestyle, including the adoption of natural and organic oral care products, seem to be the main motivation of the recent increase of “fluoride-free” tweets, which can be boosted by the propagation of fluoride falsehoods noted on the web. Therefore, public health authorities, health professionals, and legislators should be aware of the spread of fluoride-free content on social media to create and implement strategies against their potential health damage for the population.

## Appendix

Multimedia Appendix 1 Exploratory data preprocessing (term frequency filtering).

## Abbreviations

LDA: latent Dirichlet allocation

## Data Availability

The data sets generated during and/or analyzed during the current study are available from the corresponding author on reasonable request.
