# Supplementary material for: Analysis of Fluoride-Free Content on Twitter: Topic Modeling Study
Source: J Med Internet Res. 2023 Jun 20;25:e44586. doi: 10.2196/44586 (PMC10337345; doi:10.2196/44586)
Supplement: Multimedia Appendix 1 [file jmir_v25i1e44586_app1.pdf]

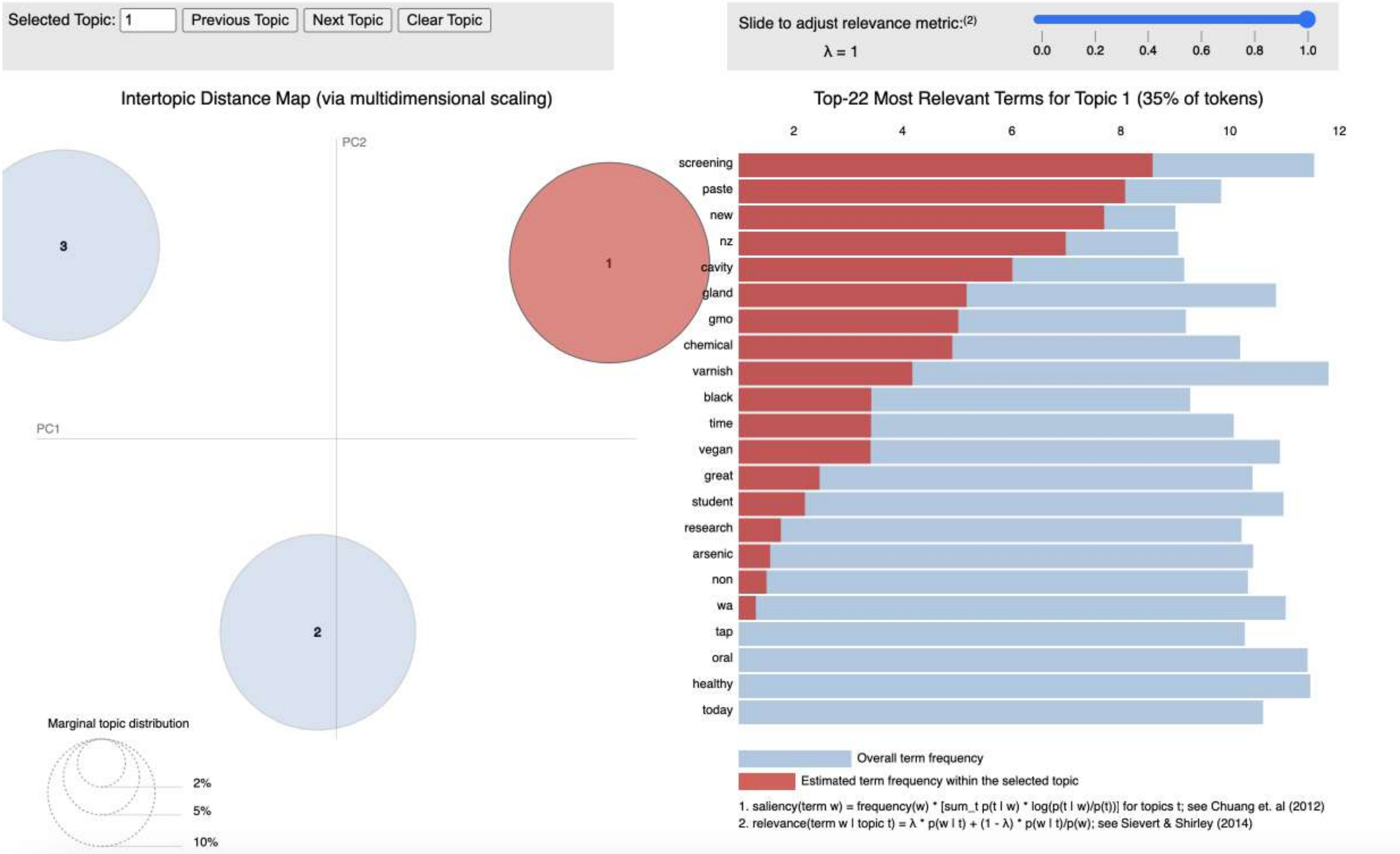

Selected Topic: 1
Previous Topic
Next Topic
Clear Topic
Slide to adjust relevance metric:(2)
λ = 1
0.0
0.2
0.4
0.6
0.8
1.0
Intertopic Distance Map (via multidimensional scaling)
PC2
PC1
1
2
3
Marginal topic distribution
2%
5%
10%
Top-22 Most Relevant Terms for Topic 1 (35% of tokens)
2
4
6
8
10
12
screening
paste
new
nz
cavity
gland
gmo
chemical
varnish
black
time
vegan
great
student
research
arsenic
non
wa
tap
oral
healthy
today
Overall term frequency
Estimated term frequency within the selected topic
1. saliency(term w) = frequency(w) * [sum_t p(t | w) * log(p(t | w)/p(t))] for topics t; see Chuang et. al (2012)
2. relevance(term w | topic t) = λ * p(w | t) + (1 - λ) * p(w | t)/p(w); see Sievert & Shirley (2014)

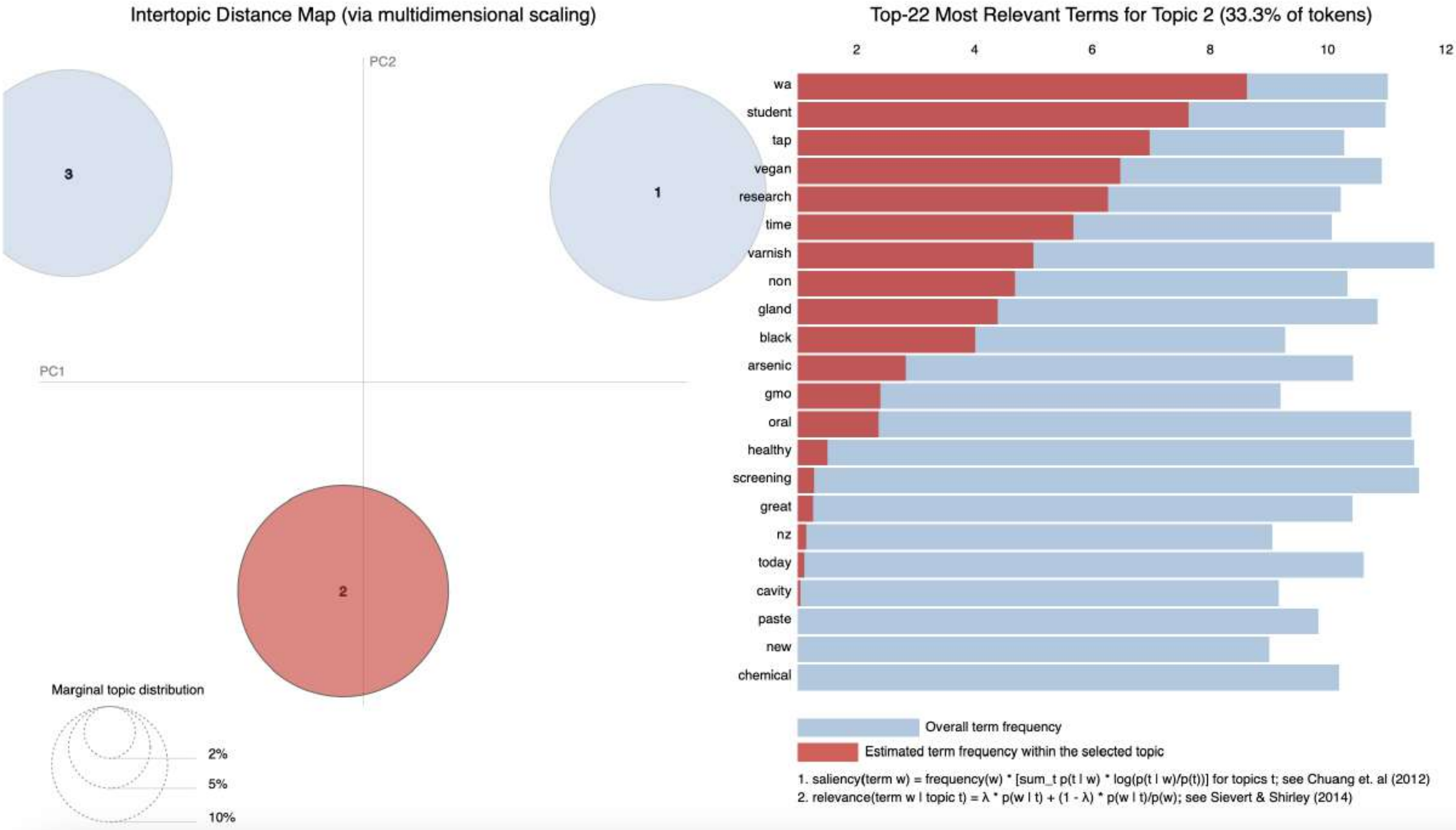
Intertopic Distance Map (via multidimensional scaling)
PC2
PC1
3
1
2
Marginal topic distribution
2%
5%
10%
Top-22 Most Relevant Terms for Topic 2 (33.3% of tokens)
2
4
6
8
10
12
wa
student
tap
vegan
research
time
varnish
non
gland
black
arsenic
gmo
oral
healthy
screening
great
nz
today
cavity
paste
new
chemical
Overall term frequency
Estimated term frequency within the selected topic
1. saliency(term w) = frequency(w) * [sum_t p(t | w) * log(p(t | w)/p(t))] for topics t; see Chuang et. al (2012)
2. relevance(term w | topic t) = λ * p(w | t) + (1 - λ) * p(w | t)/p(w); see Sievert & Shirley (2014)

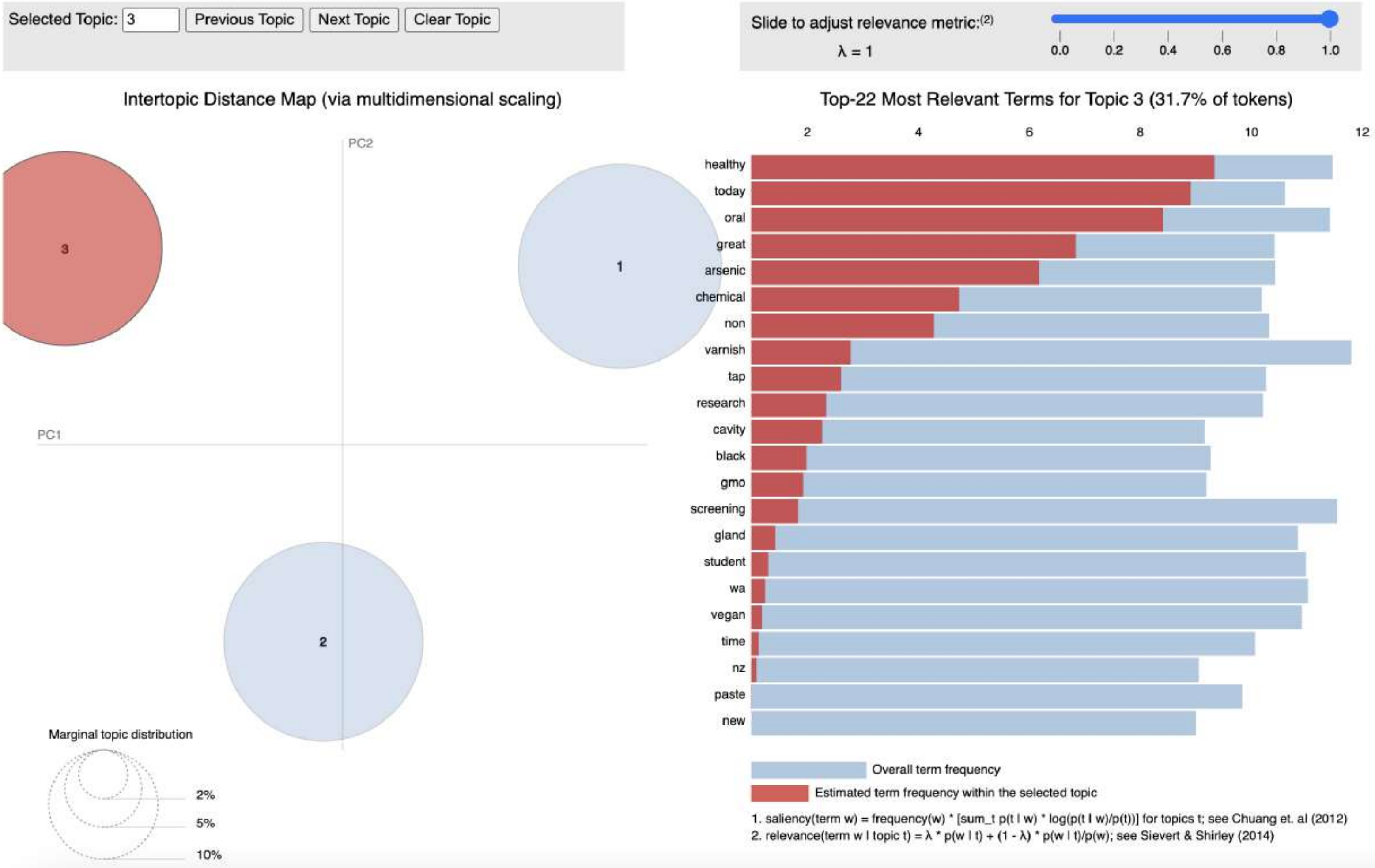
Selected Topic: 3
Previous Topic
Next Topic
Clear Topic
Slide to adjust relevance metric:(2)
λ = 1
0.0
0.2
0.4
0.6
0.8
1.0
Intertopic Distance Map (via multidimensional scaling)
PC2
PC1
1
2
3
Marginal topic distribution
2%
5%
10%
Top-22 Most Relevant Terms for Topic 3 (31.7% of tokens)
2
4
6
8
10
12
healthy
today
oral
great
arsenic
chemical
non
varnish
tap
research
cavity
black
gmo
screening
gland
student
wa
vegan
time
nz
paste
new
Overall term frequency
Estimated term frequency within the selected topic
1. saliency(term w) = frequency(w) * [sum_t p(t | w) * log(p(t | w)/p(t))] for topics t; see Chuang et. al (2012)
2. relevance(term w | topic t) = λ * p(w | t) + (1 - λ) * p(w | t)/p(w); see Sievert & Shirley (2014)
